# Surgical treatment for secondary aortoesophageal fistula

**DOI:** 10.1186/s13019-020-01293-x

**Published:** 2020-09-11

**Authors:** Kayo Sugiyama, Toru Iwahashi, Nobusato Koizumi, Toshiya Nishibe, Toshiki Fujiyoshi, Hitoshi Ogino

**Affiliations:** 1 Department of Cardiac Surgery, Aichi Medical University, 1-1 Yazakokarimata, Nagakute, Aichi 480-1195 Japan; 2 Department of Cardiovascular Surgery, Tokyo Medical University, 6-7-1 Nishishinjuku, Shinjuku-ku, Tokyo, 160-0023 Japan

**Keywords:** Secondary aortoesophageal fistula, Thoracic endovascular aortic repair, Total arch replacement

## Abstract

**Background:**

Aortoesophageal fistula (AEF) is a relatively rare condition that is often life-threatening. Secondary AEF is a complication of previous surgery, which can be more critical and challenging than primary AEF. The number of secondary AEF is increasing due to increase in the number of thoracic endovascular aortic repair (TEVAR). Although TEVAR has become a successful alternative surgical strategy for thoracic aortic aneurysms, secondary AEF after TEVAR might be critical than other secondary AEF because of severe adhesion between the esophagus and residual thoracic aortic wall.

**Methods:**

This study analyzed six patients with secondary AEF who were treated at Tokyo Medical University Hospital between 2011 and 2016. These participants included four patients who had undergone TEVAR and two who had undergone total arch replacement.

**Results:**

Although they were subsequently hospitalized for a long period, open surgical repair was completed in two patients who had undergone total arch replacement. TEVAR alone was performed in two patients who had undergone TEVAR and they were discharged without major complications shortly. Combined repair of TEVAR as a bridge to open surgery was planned for two patients who had undergone TEVAR. However, reconstruction of the aorta and esophagus could not be completed in these patients due to severe adhesions, and they died during hospitalization.

**Conclusions:**

Definitive open repair was successfully performed in patients with secondary AEF after total arch replacement. However, in the patients with secondary AEF after TEVAR, severe adhesion between the aorta and esophagus led to difficulty in performing a successful definitive open repair. The strategy for secondary AEF should, therefore, be decided considering the etiology of secondary AEF. In secondary AEF after TEVAR, definitive open repair is difficult to complete because of catastrophic complication, and palliative treatment using TEVAR without reconstruction of aorta and esophagus can be an alternative.

## Background

Aortoesophageal fistula (AEF) is a relatively rare condition that is often life-threatening [1–3]. Secondary AEF, a complication of surgery in the posterior mediastinum can be more critical than primary AEF because of the presence of adhesion in the pleural space and the poor general status of patients. The number of secondary AEF is increasing due to increase in the number of TEVAR. There have been few studies comparing the surgical results for secondary AEF cases [4, 5] and optimal strategy in management of secondary AEF is still controversial.

## Methods

We retrospectively reviewed the clinical charts of six patients who required surgical intervention for the treatment of secondary AEF at Tokyo Medical University Hospital between 2011 and 2016. Primary AEF resulting from esophageal malignancy, thoracic aortic aneurysm, foreign body ingestion, prolonged gastric tube intubation and etc. was excluded. Diagnoses were made based on enhanced computed tomography and endoscopic examination. According to the results of blood cultures, antibiotics were initiated based on the recommendations of the infection control team in our institute. This study was conducted in compliance with the Declaration of Helsinki. All patients provided written informed consent for using their clinical data for scientific presentations or publications.

**Fig. 1:**
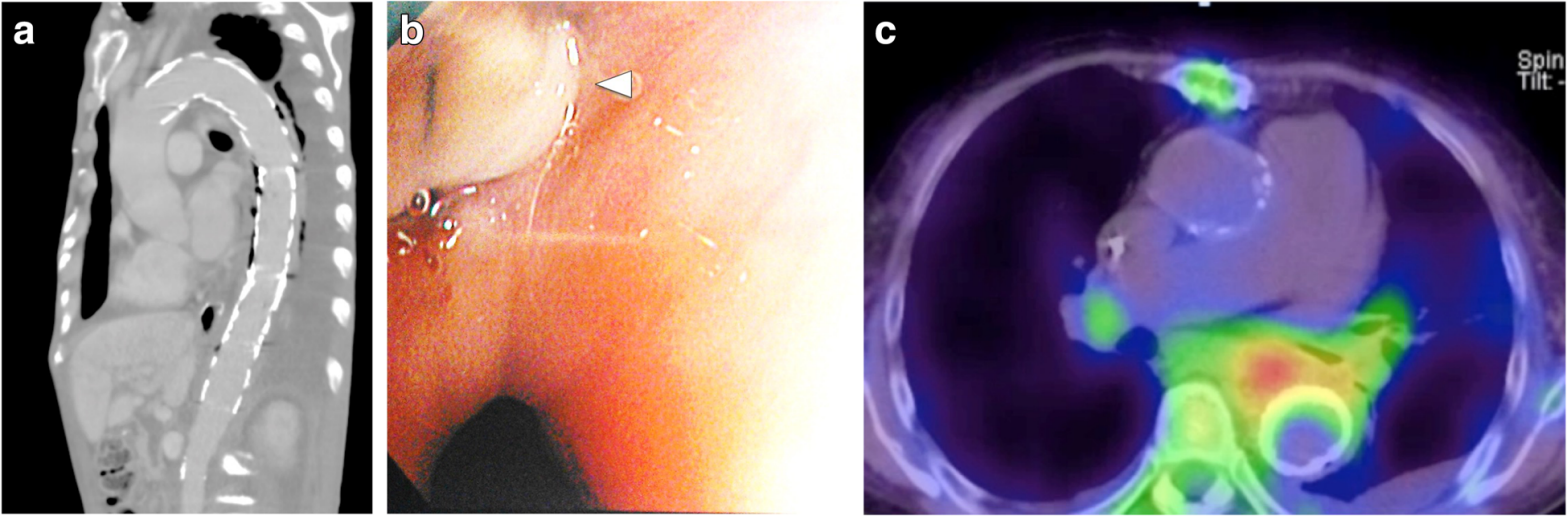
**a** Preoperative computed tomography scan of a combined repair case demonstrating massive air bubbles around the previous stent-graft. **b**: Endoscopic view showing exposure of the sutures and pledget (white arrow head) of the previous graft. **c**: Preoperative gallium scintigraphy demonstrating enhancement between the aorta and esophagus

The surgical treatment strategy for each case was designed based on the patient’s frailty and infection severity [1–3]. Treatment strategies were considered by a multidisciplinary team that included cardiac and vascular surgeons, general surgeons, radiologists, and anesthesiologists. Optional treatment strategies for AEF included open surgical repair, thoracic endovascular aortic repair (TEVAR) alone, and combined repair with TEVAR and open surgery. In patients with severe infections indicated by air bubbles between the aorta and esophagus on computed tomography (Fig. 1a) or an obvious fistula on endoscopy (Fig. 1b), open surgical repair was considered. In severely deteriorated patients, TEVAR alone was considered. Deteriorated patients without severe infection as described above chose TEVAR alone sterategy. However, due to fears of recurrent infections and complications of this palliative treatment, this strategy was carefully selected. In severely deteriorated patients with severe infection, combined repair with TEVAR as a bridge to open surgery was considered.

**Fig. 2:**
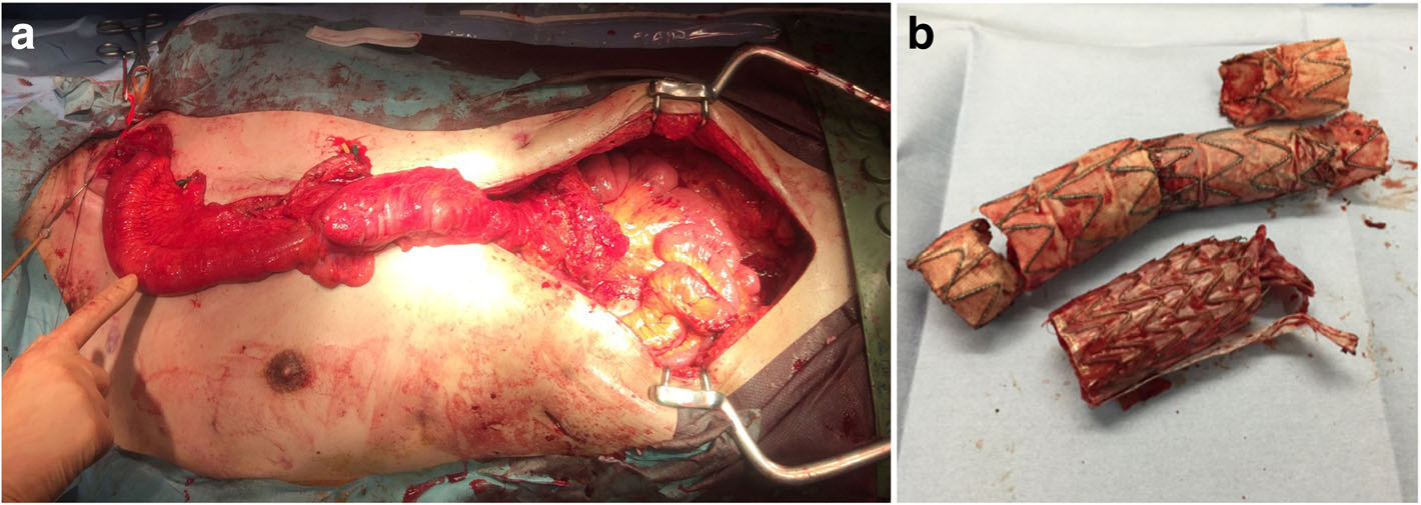
**a**: Intra-operative photograph showing reconstruction of the esophagus using the terminal ileum and right colon. **b**: The resected stent-graft in a combined repair case that was treated with an extra-anatomical bypass

Open surgical repair was performed in stages. Following esophagectomy, the proximal stump of the esophagus was pulled out to the left side of the neck and an esophagostomy was performed. Then, reconstruction of the descending aorta using a rifampicin-soaked Dacron graft was performed. After aortic reconstruction, the pleural cavity was left open and copiously irrigated with 12 to 24 l of 0.2% Gentian violet solution [3] per day for about 3 days. After then, omental wrapping around the artificial graft in the left pleural space was performed and the chest was closed. After several months, when the patient’s condition had stabilized, reconstruction of the esophagus using colon interposition was performed through the anterosternal route (Fig. 2a). Combined repair was also planned in stages. Following TEVAR to control bleeding due to hematemesis and gain hemodynamic stability, open debridement and reconstruction of esophagus and aorta were planned. In all TEVAR procedures, GORE® TAG® Thoracic Stent Graft (Gore & Associates, Flagstaff, AZ) was chosen as stent-graft because of the durability to infection of expanded polytetrafluoroethylene. Open surgical repair was done by one experienced cardiac surgeon and TEVAR was done by one experienced vascular surgeon.

We assessed clinical outcomes including complications of early and late stages. The early and late mortalities, cause of death, major adverse aortic events and recurrence of infection were also evaluated. Feasibility of the treatment strategy was considered based on these outcomes.

## Results

Patient characteristics, management strategies, and treatment outcomes are summarized in Table 1. The mean age was 67 years (range, 41–78 years); five patients were male (83%). Four patients had undergone TEVAR, two for thoracic aortic aneurysm, and two for chronic aortic dissection. Three (75%) of the four post-TEVAR cases underwent emergency TEVAR for impending rupture of the thoracic aorta. Two patients had undergone total arch replacement, one for thoracic aortic aneurysm, and one for chronic aortic dissection. There were no patients who had previously undergone esophageal surgery.

**Table 1:**
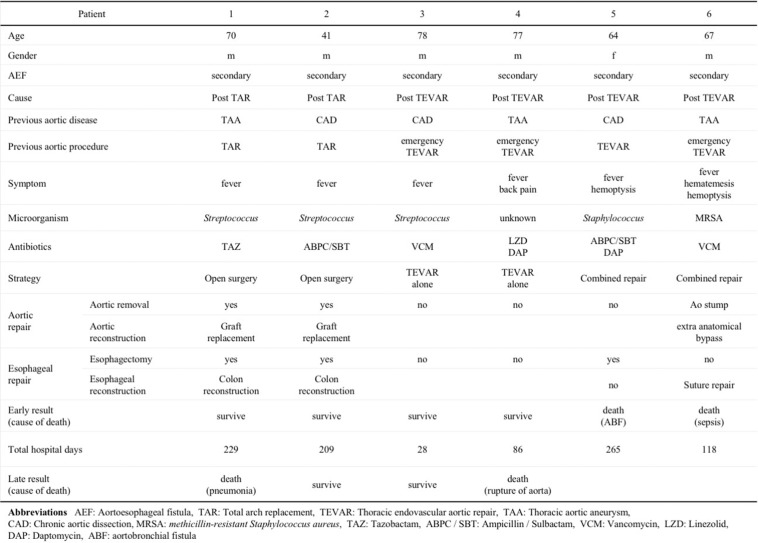
Patient characteristics, surgical methods, and outcomes

All six patients presented with fever and complained of general fatigue. The causative bacteria were detected in five patients (83%); *Streptococcus* in three and *Staphylococcus* in two. Antibiotics were administered preoperatively and continued for at least 4 weeks following surgery. Gallium scintigraphy successfully detected the primary origin of sepsis in four patients (Fig. 1c). Computed tomography was performed in all patients and additional endoscopy was performed in five (83%) patients to confirm the diagnosis. In three patients, computed tomography demonstrated severe infection with air bubbles between the aorta and esophagus (Fig. 1a). In two patients, endoscopy clearly identified the fistulas (Fig. 1b).

Two patients who had undergone total arch replacement underwent definitive open surgical repair (Fig. 2a); of the patients who had undergone TEVAR previously, two underwent TEVAR alone and two underwent combined repair (Fig. 2b). In the patients who had open surgical repair (Fig. 2a), a longer period until treatment completion was necessary (229 days and 209 days), but they were discharged without complications. It took several months to complete the treatment of creating an anterosternal route by balloon dilation. Meanwhile, they continued to take liquid nutrition at home. The patients who underwent TEVAR alone had required urgent surgery to control bleeding, and TEVAR was, therefore, performed without esophagectomy or reconstruction of the aorta. These patients were discharged without major complications shortly following surgery, in 28 and 86 days. In one of the two patients who underwent combined repair, the aorta could not be resected due to severe adhesions, and esophageal reconstruction could not be completed following esophagectomy due to the severely deteriorated state of the patient. This patient died of an aortobronchial fistula during hospitalization. In the other patient, an additional extra-anatomical bypass from the ascending aorta to the abdominal aorta with closure of the aortic stump was performed due to severe adhesions (Fig. 2b) and esophageal repair with plain suturing was performed without esophagectomy. This patient died of sepsis during hospitalization.

Overall, in-hospital mortality occurred in two (33%) of the total 6 patients, who underwent combined repair. The condition of these patients was too deteriorated to undergo definitive repair of the aorta and esophagus, and they were subsequently hospitalized for a long period (118 days and 265 days). The reasons for long-term hospitalization are due to their frailty and the difficulty of controlling the infection. Continued hospitalization is unavoidable due to the administration of antibiotic agents. In addition, oral nutrition was difficult due to the esophageal disease. This led to further vulnerability and reduced resistance to infection. Furthermore, they had severe adhesions between the aorta and esophagus. Late mortality occurred in two (50%) of the remained four patients. One patient who underwent TEVAR alone died of rupture of the remaining thoracic aortic aneurysm 6 months following surgery, and one patient who underwent surgical open repair died of pneumonia 1 year following surgery.

## Discussion

AEF is a relatively rare condition that is often life-threatening [1–3]. There are several etiologies of primary AEF including thoracic aortic aneurysm, foreign body ingestion, esophageal malignancy, and prolonged gastric intubation [6, 7]. There are several etiologies of secondary AEF resulting from surgery in the posterior mediastinum such as aortic arch replacement, TEVAR to descending aorta, and esophageal surgery [4, 8]. The number of secondary AEF is increasing due to increase in the number of TEVAR being performed. Treatment of secondary AEF can be more challenging than that of primary AEF due to the presence of adhesion in the pleural space and the poor general status of patients. In the patients in this study who underwent combined repair, reconstruction of the aorta and esophagus could not be completed due to severe adhesions caused by previous TEVAR. There have been few studies comparing the degree of adhesion between post-TEVAR cases and post-graft replacement cases, however, secondary AEF after TEVAR can be more critical than secondary AEF after graft replacement.

The exact mechanism of secondary AEF after TEVAR remains unknown. Eggebrecht et al. reported that the incidence of AEF was 1.9% and occurred 1–16 months following intervention in a series of 268 patients who underwent TEVAR [5]. Some reports suggested that its pathogenesis was related to inflammation of the aneurysmal wal [9, 10]. They also suggested that the pathogenesis was related to esophageal ischemia secondary to elevated pressure in the posterior mediastinum, inflammation due to the resorbed hematoma, and mechanical compression by a large aneurysm following TEVAR [9, 10]. In post-TEVAR cases, fistulas were reportedly caused by endoleaks into the residual aneurysmal sac, erosion of the stent-graft through the aorta, and ischemic necrosis of the esophageal wall due to compression of its feeding arteries by the stent-graft [4, 5]. In accordance with these reports, severe adhesion between the descending aorta and esophagus might occur more frequently in secondary AEF after TEVAR than after graft replacement. In aortic rupture cases, endovascular stenting does not remove the hematoma or the thrombosis, and thoracic compartment syndrome is likely to occur. Therefore, emergency TEVAR is associated with an increased risk of AEF occurrence [4, 5, 11]. In the present study, in three (75%) patients who had undergone TEVAR previously, AEF developed after emergency TEVAR.

It has been previously reported that aggressive treatment for patients with AEF was associated with good outcomes [5, 10, 12] and reduced short-term mortality. In the present study, although patients who received open surgical repair were hospitalized for a longer period, they were discharged without complications. However, the patients who underwent TEVAR alone were also discharged without complications. Moreover, they were hospitalized more shortly than open surgical repair cases. The patients who received combined repair were hospitalized for a long period and died subsequently during hospitalization. They were forced to stay in the hospital for a long time because of catastrophic complications including difficulty of controlling the infection and malnutrition due to esophageal disease. In secondary AEF after TEVAR cases, palliative treatment using TEVAR without open reconstuction of aorta and esophagus may be better as an alternative. If the remained esophagus is problematic or bleeding control is necessary, esophageal stent can be introduced. Although TEVAR was proposed as an alternative surgical management strategy for open surgery [13], late complications of TEVAR are becoming increasingly evident [4, 5]. The treatment strategy for secondary AEF after TEVAR should be carefully designed considering the presence of severe adhesions between the aorta and esophagus. Moreover, the patient’s frailty should be considered in the decision of treatment. Further studies on treatment strategy for secondary AEF including surgical indications and exclusion criteria are warranted.

This study had several limitations. First, a small number of patients was included owing to the rarity of secondary AEF. Therefore, accurate evaluation might be difficult because combined surgery was performed on severely ill patients. Second, it was a retrospective study and the data were obtained from a single institution. Therefore, the study results may not reflect the general features of patients with AEF. Third, endovascular treatment strategy has evolved during the time period of this study.

## Conclusions

Previous TEVAR may cause severe adhesions between the aorta and esophagus which may result in secondary AEF and make definitive repair of this condition very difficult. In secondary AEF cases, the surgical treatment strategy should be decided depending on each patients’ status and the etiology of the fistula. In secondary AEF after open repair, definitive open repair may be feasible, however, in secondary AEF after TEVAR, definitive open repair is difficult to complete because of catastrophic complication, and palliative treatment using TEVAR without reconstruction of aorta and esophagus can be an alternative.

## Data Availability

Not applicable.

## Abbreviations

AEF: Aortoesophageal fistula
TEVAR: Thoracic endovascular aortic repair
